# Discovery of SARS-CoV-2 Nsp14 and Nsp16 Methyltransferase Inhibitors by High-Throughput Virtual Screening

**DOI:** 10.3390/ph14121243

**Published:** 2021-11-30

**Authors:** Raitis Bobrovs, Iveta Kanepe, Nauris Narvaiss, Liene Patetko, Gints Kalnins, Mihails Sisovs, Anna L. Bula, Solveiga Grinberga, Martins Boroduskis, Anna Ramata-Stunda, Nils Rostoks, Aigars Jirgensons, Kaspars Tars, Kristaps Jaudzems

**Affiliations:** 1 Latvian Institute of Organic Synthesis, Aizkraukles 21, LV-1006 Riga, Latvia; iveta@farm.osi.lv (I.K.); nauris.narvaiss@osi.lv (N.N.); anna.lina.bula@osi.lv (A.L.B.); solveiga@osi.lv (S.G.); aigars@osi.lv (A.J.); kristaps.jaudzems@osi.lv (K.J.); 2 Faculty of Biology, University of Latvia, Jelgavas 1, LV-1004 Riga, Latvia; liene.patetko@gmail.com (L.P.); martins.boroduskis@lu.lv (M.B.); anna.ramata-stunda@lu.lv (A.R.-S.); nils.rostoks@lu.lv (N.R.); 3 Latvian Biomedical Research and Study Centre, Ratsupites 1 k1, LV-1067 Riga, Latvia; gints.kalnins@biomed.lu.lv (G.K.); mihails.shishovs@gmail.com (M.S.); kaspars@biomed.lu.lv (K.T.)

**Keywords:** SARS-CoV-2, MTase inhibitors, nsp14, nsp16, antiviral drugs, high-throughput virtual screening

## Abstract

The severe acute respiratory syndrome coronavirus 2 (SARS-CoV-2) uses mRNA capping to evade the human immune system. The cap formation is performed by the SARS-CoV-2 mRNA cap methyltransferases (MTases) nsp14 and nsp16, which are emerging targets for the development of broad-spectrum antiviral agents. Here, we report results from high-throughput virtual screening against these two enzymes. The docking of seven million commercially available drug-like compounds and S-adenosylmethionine (SAM) co-substrate analogues against both MTases resulted in 80 virtual screening hits (39 against nsp14 and 41 against nsp16), which were purchased and tested using an enzymatic homogeneous time-resolved fluorescent energy transfer (HTRF) assay. Nine compounds showed micromolar inhibition activity (IC_50_ < 200 μM). The selectivity of the identified inhibitors was evaluated by cross-checking their activity against human glycine N-methyltransferase. The majority of the compounds showed poor selectivity for a specific MTase, no cytotoxic effects, and rather poor cell permeability. Nevertheless, the identified compounds represent good starting points that have the potential to be developed into efficient viral MTase inhibitors.

## 1. Introduction

Coronaviruses (CoVs) known to infect humans have caused three major disease outbreaks within the past two decades. Severe acute respiratory syndrome (SARS) CoV caused the SARS epidemic from 2002 to 2004; the Middle East respiratory syndrome (MERS) CoV caused outbreaks in 2012, 2015, and 2018; and SARS-CoV-2 caused the global CoV disease 2019 pandemic (COVID-19) [1,2]. The genome of the CoVs contains a replicase polyprotein region referred to as Open Reading Frame 1a and 1b (ORF1a,b), which encodes the non-structural proteins (nsps), and a structural region, which encodes spike, envelope, membrane, nucleocapsid proteins, and several accessory genes [3]. The enzymatic activities and functional domains of the nsps are conserved between the various CoVs, affirming their significance in the viral life cycle [4]. Some of the nsps are particularly well conserved between CoVs, such as nsps 11, 12, 13, 14, and 16 [3]. The high homology between these enzymes in various CoVs makes them attractive drug targets, as compounds targeting an enzyme of one CoV would most likely show activity against other CoVs as well. Such drugs would allow combating newly emerging CoVs in the future much more rapidly.

**Figure 1:**
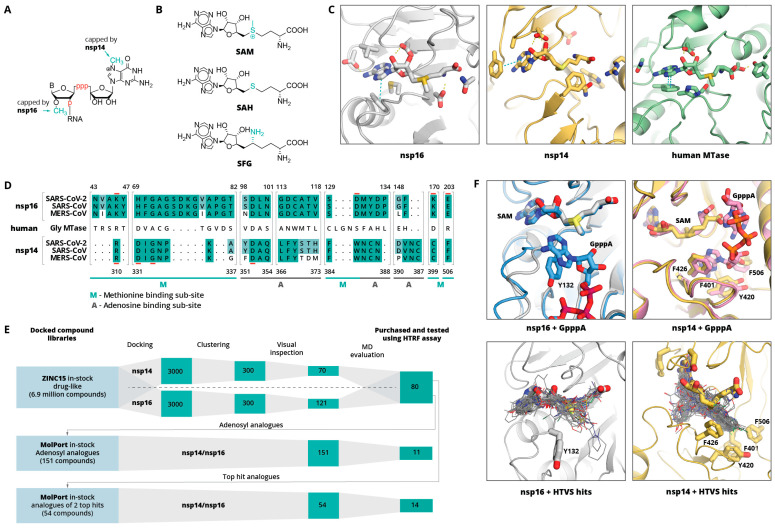
(**A**) Schematic representation of nsp14 and nsp16 mRNA capping sites. B indicates base, p indicates phosphate group. (**B**) Molecular structures of methyl group donor S-adenosylmethionine (SAM), the reaction product S-adenosylhomocysteine (SAH), and the SAH analogue sinefungin (SFG). (**C**) Binding sites of SARS-CoV-2 nps16 (PDB ID: 6W4H), SARS-CoV nsp14 (PDB ID: 5C8T), and human glycine N-methyltransferase (PBD ID: 1R74 [38]). Bound SAM and key binding site residues are shown as sticks. Yellow dashed lines indicate hydrogen bonds, cyan—aromatic stacking. (**D**) Sequence alignment of nsp14 and nsp16 binding sites of SARS-CoV-2, SARS-CoV, and MERS CoV. Shading indicates the conservation of residues with fully conserved positions shaded in darker green. An aligned sequence of the human glycine N-methyltransferase is given in the middle. Residue numbers on top refer to the SARS-CoV-2 nsp16 and on the bottom refer to SARS-CoV nsp14. Active site residues are underlined in red. The adenine and methionine fragment binding sub-sites are indicated with A or M, respectively. (**E**) Schematic workflow of the computational inhibitor discovery approach used. Number of compounds retained at each stage is shown in green rectangles. The workflow was carried out for both targets separately, and the identified virtual screening hits were tested experimentally against both enzymes. (**F**) Structural alignment of GpppA-bound and unbound nsp14 and nsp16 complexes with SAM (top) and 50 top-scoring HTVS hits (bottom). SAM, GpppA, and hydrophobic amino acids interacting with RNA base are shown as sticks, HTVS hits are shown as lines.

Nsp14 and nsp16 are involved in viral mRNA capping (Figure 1A), which is a tactic used by many RNA viruses to avoid recognition by the host immune system. Nsp14 is a bifunctional enzyme with independent guanine-N^7^-methyltransferase (N^7^-MTase) and exoribonuclease (ExoN) domains [5], whereas nsp16 is a 7-methylguanine-triphosphate-adenosine (m^7^GpppA) specific 2′-O- methyltransferase (2′-O-MTase). Both enzymes use S-adenosylmethionine (SAM) as a methyl group donor (Figure 1B, C). Moreover, both of these enzymes associate with nsp10, which is necessary for the nsp16 MTase activity, and the nsp14 exoribonuclease activity (it does not have an effect on the nsp14 MTase activity) [6]. Nsp10 is a stable monomeric protein that does not have specific enzymatic activity; however, it is known to bind zinc and RNA [6,7,8]. The mutation or inhibition of nsp14 and/or nsp16 has been shown to lead to a significantly attenuated virus that is recognised by the innate immune system [9,10,11].

Up until now, MTases have been targeted to fight various cancers, and numerous MTase inhibitors have been reported, most notably, lysine MTases EZH2/EZH1 and histone MTase disrupter of telomeric silencing 1-like (DOT1L) inhibitors [12,13,14,15,16,17,18]. Some of the MTase inhibitors have reached the clinical trials [19,20]. The diversity of known MTase inhibitors, especially CoV MTase inhibitors, is limited, as the majority of them are adenosyl group containing compounds (SAM analogues) [21,22,23,24]. In addition, these compounds usually do not exhibit particularly good selectivity for viral over human MTases, and they display poor cell permeability due to their high polarity and/or zwitterionic nature [25,26,27,28]. Recently, there have been several attempts to identify SARS-CoV-2 MTase inhibitors that are not adenosyl analogues [15,29,30,31,32,33,34,35,36]; however, their activity has not always been proven experimentally.

Here, we aim to identify novel drug-like compound classes that inhibit the SARS-CoV-2 MTases nsp14 and nsp16 and could serve as starting points for lead optimisation. New inhibitors were identified via high-throughput virtual screening (HTVS) of a wide chemotype space compound library. Molecular docking was enabled by the availability of several SARS-CoV-2 nsp16/nsp10 heterodimer crystal structures [5] in complex with its methyl group donor S-adenosylmethionine (SAM), the reaction product S-adenosylhomocysteine (SAH), and the SAH analogue sinefungin (SFG). SARS-CoV-2 nsp14, on the other hand, does not have any crystal structure. However, 95% identity (99% similarity) to SARS-CoV (100% identity in the binding site, Figure 1D), allows using its structure [37] for structure-based drug discovery.

## 2. Results and Discussion

Figure 1E shows a schematic workflow of our approach used for hit discovery against SARS-CoV-2 nsp14 and nsp16. We started our study by docking model validation, where the known MTase adenosyl group-containing compounds (SAM, SAH, and SFG) were docked alongside 100 property-matched decoys (generated using DUD-E [39]). Models that returned SAM analogues in the correct docked pose and amongst the top scoring compounds were selected for further studies. An HTVS campaign was initiated by docking the ZINC15 in-stock drug-like compound library [40] (6.9 million compounds) into SARS-CoV-2 nsp16 (PDB 6W4H) and SARS-CoV nsp14 (PDB 5C8T) crystal structures (see Supplementary Material, Methods Section). To retain only the unique virtual screening hit scaffolds (i.e., the top scoring compound from each cluster), top scoring 3000 compounds for each enzyme were clustered by applying Tanimoto similarity metrics to linear molecule fingerprints. The top ranked 300 compounds for each enzyme were visually inspected for their ability to form hydrogen bonds similar to SAM, and molecules showing internal strains or unsatisfied hydrogen bond donors were deprioritised. Ultimately, 70 potential nsp14 and 121 nsp16 binders were promoted to molecular dynamics (MD) studies. The stability of the docked binding pose for each enzyme/compound complex was evaluated by running 10 parallel 20 ns MD simulations and calculating the ligand RMSD throughout the simulation against the docked pose. Compounds that retained the docked pose (RMSD <2 Å) in at least seven parallel MD runs were purchased from commercial vendors. This approach resulted in a list of 80 highly diverse compounds (39 potential nsp14 and 41 nsp16 binders; see Table S1).

Despite the poor binding site sequence similarity between nsp14 and nsp16 (Figure 1D), the shape of the binding site and charge distribution is similar. Therefore, while the identified virtual screening hits showed great diversity, there were several structural similarities between the majority of the compounds. Most of the compounds had either two or three aromatic rings, where one of them occupied the adenine binding sub-site, while another one bound in either the amino acid binding sub-site or a hydrophobic mRNA binding sub-pocket. In the adenine binding sub-site, the confirmed HTVS hits were involved in interactions similar to those of SAM—aromatic stacking with Phe367 (nsp14) and Phe149 (nsp16), and hydrogen bonding with Tyr368 backbone amide group nitrogen (nsp14) and Cys115 amide and thiol groups (nsp14). The interactions between the aspartate typically involved in hydrogen bonding with the ribose group (Asp352 in nsp14 and Asp99 in nsp16) and HTVS hits were suboptimal and could be a location for further ligand optimisation. The most pronounced binding site differences are in the methionine binding sub-site, and it is believed that selectivity could be achieved by targeting this region. Here, HTVS hits mostly interact with nsp14 Asn388 amide groups via hydrogen bonds and/or Phe426 via aromatic stacking interactions. In the case of nsp16, the key interactions are with the Tyr132 main chain. Surprisingly, none of the verified HTVS hits interacted with charged amino acids in the methionine binding sub-site similarly to the SAM carboxylic group. The introduction of a functional group that is able to form such interactions could greatly increase the activity of compounds and by maintaining already formed interactions, hopefully, specificity.

The HTVS hit aromatic rings that bound in the amino acid binding sub-site was in most cases a heterocycle that mimicked the carboxylic group, whereas the aromatic groups that bound in the hydrophobic mRNA sub-pocket were structurally more diverse. Binding in the terminal guanosine sub-pocket was particularly pronounced for nsp14 hits, which formed interactions with residues Phe401, Tyr420, Phe426, and Phe506 (Figure 1F). It is worth noting that this rather enclosed, hydrophobic mRNA base binding pocket is maintained even when no mRNA is bound (Figure 1F). Binding in the terminal guanosine sub-pocket was not observed for the nsp16 hits, as this sub-site is much wider and shallower; thus, ligand substituent interacting with the aromatic group of Tyr132 would be solvent exposed. In addition, a comparison of the nsp16 structures with and without bound GpppA suggests that this sub-pocket is more flexible than that of nsp14. While the main aim of the studies was to identify new drug-like compounds, some of the top-scoring HTVS hits were already known MTase binders—adenosyl analogues, such as SAM and SFG.

It has been shown that inhibitory activity of the cap MTase inhibitors can be determined using radioactive MTase assay [21,41], LC-MS based MTase assay [41], and homogeneous time-resolved fluorescent energy transfer (HTRF) assay [14]. Here, the inhibitory activity of the SARS-CoV-2 MTase virtual screening hits was evaluated in an HTRF assay, which is based on the quantification of released S-adenosyl homocysteine (SAH) during the enzymatic reaction. Since both MTases use the same molecule (SAM) as a methyl group donor, and potential inhibitors were identified by docking into the SAM binding site, the potential nsp14 inhibitors were cross-checked against nsp16 and vice versa. The selectivity of the most potent compounds was evaluated by measuring their potency against human glycine N-methyltransferase (hGNMT), a highly abundant SAM-dependent human enzyme, which competes with tRNA methyltransferases for SAM and regulates the relative levels of SAM and SAH in cells [42].

**Figure 2:**
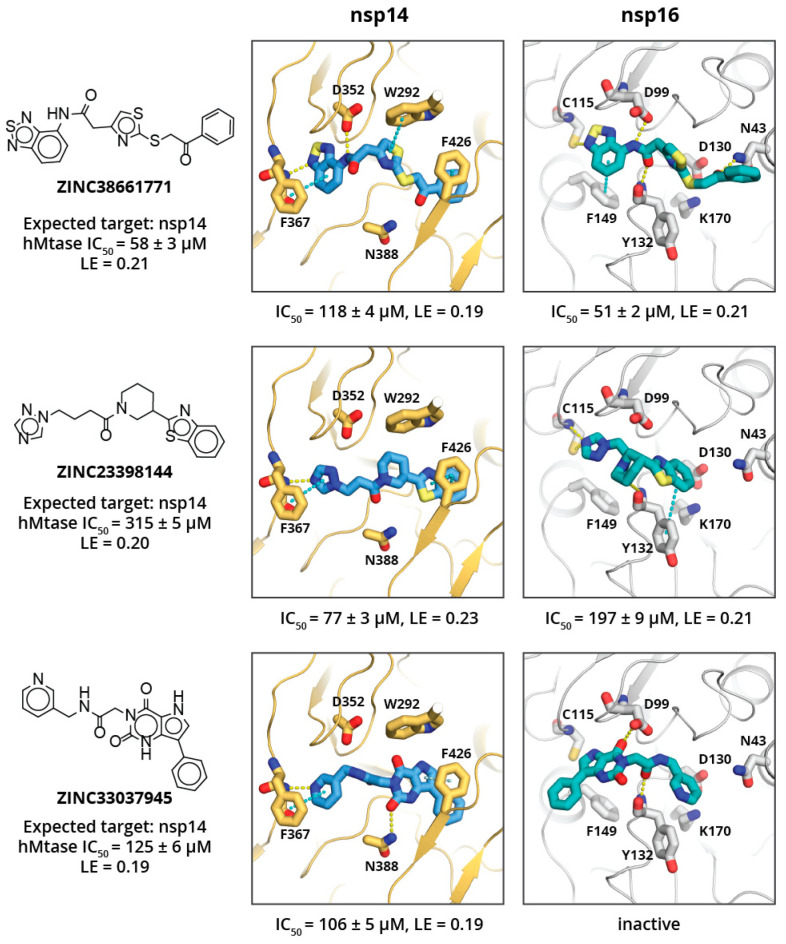
Virtual screening hits against nsp14 and nsp16. Compound molecular structure, ZINC ID, target MTase, IC_50_ activity (μM), and ligand efficiency (LE, kcal/mol) against human glycine N-methyltransferase are given on the left. Docked pose of the respective compound in complex with nsp14 (centre) and nsp16 (right) with experimentally determined IC_50_ values and LE against the particular enzyme shown below. Inhibitor and key amino acid residues are shown as sticks. Yellow and cyan dashed lines indicate hydrogen bonds and aromatic stacking, respectively.

Eight of the 80 ordered compounds were not soluble in DMSO and could not be tested experimentally. The activities of the other HTVS hits were moderate, with three compounds showing IC_50_ values of ≈50–100 μM (see Figure 2), and LEs of ≈0.20 kcal/mol per non-hydrogen atom. The most potent nsp14 and nsp16 inhibitors were ZINC38661771, ZINC23398144, and ZINC33037945 (full list of tested inhibitors in Table S1). All three compounds showed no selectivity toward the viral MTases, and activities against all three tested MTases differed by two to fourfold. This, most likely, is a result of compounds fulfilling some common key interactions with all enzymes while not targeting any specific residue of a particular enzyme. The compound that showed some selectivity toward SARS-CoV-2 MTases (particularly nsp14) was ZINC23398144. Docking studies suggest that this might be a consequence of its large benzothiazole group binding in the hydrophobic mRNA binding sub-site of nsp14, which is rather shallow and solvent exposed in nsp16 and hGNMT. Another compound that showed some selectivity for nsp14 over nsp16 was ZINC33037945; however, it did not show selectivity over hGNMT. This, again, could be a result of its large, three aromatic ring system binding in the mRNA binding sub-site of nsp14 or spacious methionine binding site of the hGNMT, and inability to fit in the mRNA sub-pocket of nsp16.

The binding site analysis suggest that compound selectivity could be improved by restricting ligand conformation freedom and/or targeting both hydrophobic mRNA and methionine binding sub-sites simultaneously, since the relative position of these sub-pockets is different for each enzyme. The HTVS hits identified here show high flexibility and therefore are able to occupy conformations that allow binding to various MTases, thus showing poor selectivity.

The three most potent compounds were further characterised for cytotoxicity and cell permeability (against 3T3, A549, and HepG2 cell lines), which is a common issue with nucleoside derivatives [25,26,27,28]. Compounds ZINC23398144 and ZINC33037945 exhibited no measurable cytotoxicity, while ZINC38661771 showed cytotoxicity at ≈ 100 μM concentration. Compound ZINC33037945 also exhibited good cell permeability, while compounds ZINC38661771 and ZINC23398144 showed a low level of cell permeability (see Table 1).

Since identified SARS-CoV-2 MTase inhibitors are rather small molecules with acceptable LEs and show drug-like properties, they can serve as templates for further chemical elaboration into lead-like molecules. To explore the chemical space around these compounds, and gain additional insight on the potential compound development route, commercially available hit analogues were purchased. Additionally, for comparison with the current best-in-class, commercially available SAM analogues that are well known to inhibit MTases were also purchased.

**Figure 3:**
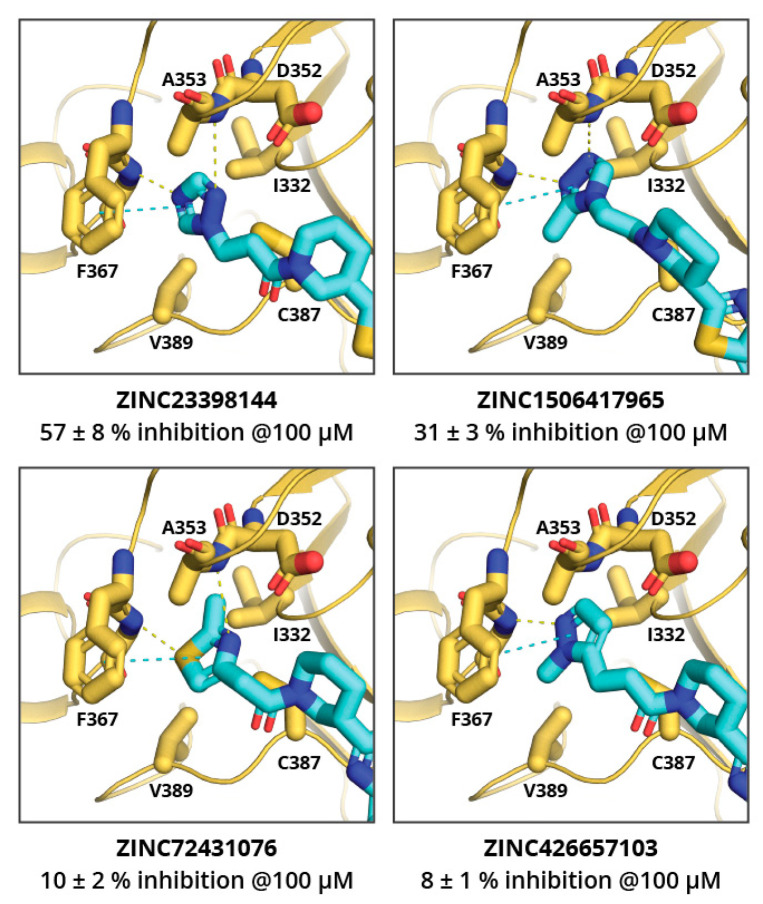
Binding poses of several active ZINC23398144 analogues docked in nsp14 adenine binding sub-pocket. Compound ZINC ID and percent inhibition of nsp14 at 100 μM concentration is given bellow. Ligand and key amino acid residues are shown as sticks. Yellow dashed lines indicate hydrogen bonds; cyan indicate aromatic stacking.

The MolPort in-stock compound database was screened for ZINC23398144 and ZINC38661771 analogues, and 34 and 20 compounds, respectively, were identified. Based on the docking score and chemical diversity, 11 ZINC23398144 analogues and three ZINC38661771 analogues were purchased, and their binding potency was determined (see Tables S2 and S3). The analogues of the ZINC23398144 predominantly had chemical modifications at the triazole heterocycle, and, thus, according to molecular docking, they explored the adenine binding sub-site. Unfortunately, none of the analogues showed higher potency than the previously identified hits. ZINC23398144 analogues bearing a heterocycle as adenine replacements were expected to maintain aromatic interactions with Phe367 and hydrogen bonding with the Tyr368 main chain (Figure 3). However, other hydrogen bonding options were not introduced or maintained with analogue heterocycles, thus leading to reduced potency.

**Figure 4:**
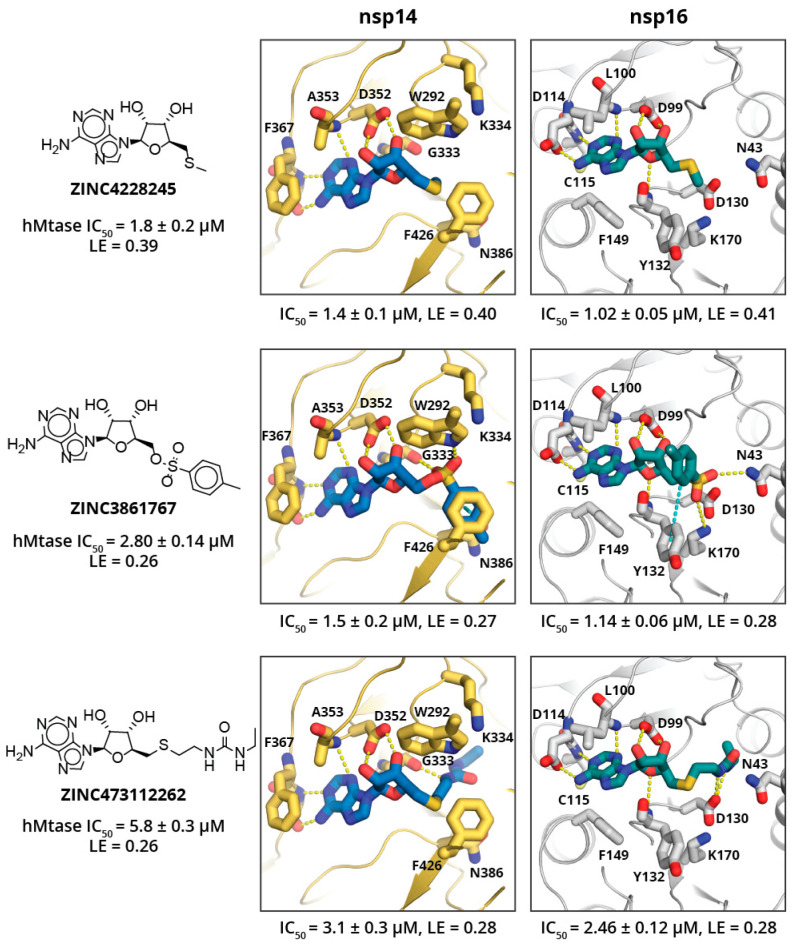
Binding poses of three most potent SAM analogues docked in nsp14 and nsp16. Compound molecular structure, ZINC ID, IC_50_ activity (μM), and ligand efficiency (LE, kcal/mol) against human glycine N-methyltransferase are given on the left. Docked pose of the respective compound in complex with nsp14 (centre) and nsp16 (right) with experimentally determined IC_50_ values and LE against the particular enzyme below. Ligand and key amino acid residues are shown as sticks. Yellow dashed lines indicate hydrogen bonds, cyan indicate aromatic stacking.

SAM analogues were extracted from the MolPort in-stock compound library (151 compounds found), docked into the target structures, and 11 chemically diverse top scoring SAM analogues were purchased from MolPort. The top scoring adenosyl group containing analogues showed activity in low μM level (see Figure 4; a full list of tested SAM analogues is in Table S4), and ligand efficiencies (LE) estimated from IC_50_ values in range from 0.27 up to 0.41 kcal/mol, which is comparable to SAH and SFG. The top-scoring SAM analogues show large structural diversity and do not provide clear SAR (see Figure 4); besides, none of the compounds show high specificity toward a particular MTase. However, this might not be surprising, as methionine substituent was replaced by a methyl group in ZINC4228245, and a methyl group that does not form particularly strong interactions with either of targets, the methylbenzenesulfonate group in ZINC3861767, is expected to interact with catalytic residues and bind in the RNA binding sub-pocket, which is rather spacious for both targets; whereas the ethylureido group in ZINC473112262 mimics the methionine and is expected to bind in the amino acid binding sub-site and occupy a pose similar to methionine. All SAM analogues tested showed no cytotoxic effects; however, this might be due to poor cell permeability (see Table 1).

## 3. Materials and Methods

### 3.1. High-Throughput Virtual Screening (HTVS)

ZINC15 in-stock drug-like compound library [40] of 6.9 million compounds was prepared using LigPrep [43] by desalting the molecules, generating possible tautomers and ionisation states at pH 7.0 ± 2.0. The stereochemistry of the compounds was retained as specified in the library.

The prepared library was docked in the crystal structure of SARS-CoV-1 nsp14 [37] (PDB ID: 5C8T) and SARS-CoV-2 nsp16 [5] (PDB ID: 6W4H) using the Schrodinger Maestro software package [44]. Protein crystal structures were prepared using Maestro Protein Preparation Wizard [43] by adding missing side chains using Prime [45], adjusting side chain protonation states at pH 7.0, and minimising heavy atoms with convergence up to 0.30 Å. Molecular docking was performed using Glide [46], with scaling of the van der Waals radii set to 0.9 for protein and ligand heavy atoms, and docking compounds flexibly. The top-scoring 3000 compounds were clustered to 300 representative compounds by calculating the Linear Fingerprints from Daylight invariant atom types and evaluating compound similarity using Tanimoto similarity metrics. The top-scoring compound was retained for each cluster. The top-ranked 300 representative compounds for each enzyme were visually inspected for their ability to form hydrogen bonds similar to SAM, with molecules showing internal strains or unsatisfied hydrogen bond donors being deprioritised. A total of 70 potential nsp14 and 121 nsp16 binders were promoted to molecular dynamics (MD) stability studies.

Analogues of the verified HTVS hits and SAM analogues were docked as described before. Library of the HTVS hit (ZINC23398144 and ZINC38661771) analogues was created by filtering the MolPort in-stock database for compounds with a Tanimoto similarity metrics coefficient above 0.7. The library of the SAM analogues was created by filtering the MolPort in-stock compound library for compounds that contain an adenosyl group. Ligands were prepared as described before.

Docked poses were visualised using PyMOL [47].

### 3.2. MD Calculations

The stability of the docked pose for the top-scoring HTVS hits was evaluated by running the MD simulations and calculating the ligand RMSD throughout the simulation against the docked pose. The MD simulation systems of selected inhibitor–enzyme complexes were prepared by placing the molecules in dodecahedral boxes with at least 1.5 nm distance to the box walls. The TIP3P water model was used to solvate the complex. Sodium and chloride ions were added to neutralise the systems and reach 150 mM salt concentration. Forcefields for the inhibitors were based on the general AMBER force field (GAFF) and were generated using Ambertools [48]. Amber03 forcefield parameters were used for protein [49,50]. The prepared systems were relaxed through an energy minimisation, which was performed using the steepest descent algorithm with a tolerance of 100 kJ/mol·nm. After minimisation, all systems were equilibrated in the NVT and then NPT ensembles for 5 ns. The MD (leapfrog) integration scheme with an integration time step of 2 fs was employed for equilibration and production runs. The particle mesh Ewald (PME) approach was used to calculate long-range electrostatic interactions with a cut-off of 0.8 nm. Both Lennard–Jones and Coulomb interactions were explicitly calculated up to 0.8 nm. The LINCS algorithm [51] was applied at each step to preserve the hydrogen bond lengths. NPT equilibration was performed employing a Berendsen barostat [52] with a coupling constant of 2 ps and reference pressure 1.0 bar. Velocity-rescale thermostat [53] with a coupling constant of 2 ps and reference temperature 298.0 K was used for equilibration and production simulations. The production runs were performed in the NPT ensemble for 20 ns, and 10 parallel simulations were performed for each complex. System coordinates were saved every 100 ps, and a total of 200 frames were generated for further analysis. The potential energy minimization and MD simulations were carried out with the software package Gromacs 2018.2 [54,55].

The stability of the docked pose was evaluated by calculating the ligand on protein RMSD value for ligand heavy atoms using Gromacs. Compound was considered as stable if the RMSD was below 2 Å for more than 90% of simulation time in at least 7 parallel runs. Stable compounds were purchased commercially. Simulations were visualised using VMD [56].

Protein expression and purification, as well as homogeneous time-resolved fluorescent energy transfer (HTRF) assay, SARS-CoV-2 nsp16/nsp10 methyltransferase substrate RNA production, cytotoxicity, and cell permeability testing was performed as described before [28].

### 3.3. Protein Expression and Purification

Nsp14 gene sequence were purchased as a gene synthesis from General Biosystems with codon optimisation for *E. coli* expression system in pET28a vector. NdeI and NotI restriction sites were used for this construct, and the construct contained an additional N-terminal His-6x tag with thrombin cleavage site. Nsp10 and nsp16 were ordered from the ATCC repository as NR-52425 and NR-52427 catalogue items, respectively. Nsp10 and nsp16 constructs were cloned in the pMCSG53 vector and contained additional N-terminal His-6x tag with tobacco etch virus protease cleavage sites.

Protein expression was performed in *E. coli* BL21-DE3 cells. Cells were maintained in LB medium supplemented with 30 μg/mL kanamycin, and the protein expression was performed in 2xTY medium supplemented with 30 μg/mL kanamycin. Cells were grown at 37 °C to OD_590_ 0.7 and shaken at 200 rpm, cooled at 20 °C for 30 min, and induced with 1 mM IPTG. Induction was performed overnight for approximately 16 h at 20 °C with shaking at 200 rpm. Biomass was collected by centrifugation and stored at −20 °C.

Procedures for nsp10, nsp14, and nsp16 purification were identical. Note that reducing conditions during purification procedures are extremely important for the stability of nsp16 and nsp14. The purification protocol was adopted from previous study [57] with modifications. Cell disruption was performed with ultrasonication in 50 mM Tris-HCl (pH 9.0), 500 mM NaCl, 10 mM 2-mercaptoethanol, 2 mM MgCl_2_, 0.1% (*v*/*v*) Triton X-100, 10% (*v*/*v*) glycerol, and 50 mM imidazole. Then, the lysate was centrifuged for 30 min at 11,000× g. The proteins were purified from lysate with IMAC using either batch or column Ni^2+^-NTA agarose. The Ni^2+^-NTA agarose were previously equilibrated in 50 mM Tris-HCl (pH 9.0), 0.5 M NaCl, 10 mM 2-mercaptoethanol, 2 mM MgCl_2_, 5% (*v*/*v*) glycerol, and 50 mM imidazole and then were washed with the same buffer. Proteins were eluted with a buffer containing 50 mM Tris-HCl (pH 9.0), 0.5 M NaCl, 10 mM 2-mercaptoethanol, 2 mM MgCl_2_, 5%(*v*/*v*) glycerol, and 1 M imidazole.

Nsp10 and nsp16 for nsp16/nsp10 assay were cleaved overnight with TEV protease by adding it to the protein solution at a ratio of 1 mg per 30 mg of cleavable protein.

Then, the proteins were further purified with size exclusion chromatography on a Superdex 200 column in 20 mM HEPES (pH 8.5), 0.5 M NaCl, 10 mM 2-mercaptoethanol, 2 mM MgCl_2_ and 5%(*v*/*v*) glycerol. Then, the peak fractions were pooled. In the case of nsp16/nsp10, a separation of uncleaved protein and TEV protease was performed at this point by passing this material through Ni^2+^-NTA agarose column.

Nsp16 and nsp10 were mixed in equimolar ratios and concentrated with ultrafiltration to a concentration of 2 mg/mL. SAM was added to a concentration of 2 mM and the mixture was incubated overnight at 4 °C to allow proper complex formation. The next day, the mixture was dialyzed against 20 mM HEPES (pH 7.5), 0.15 mol/L NaCl, 5% glycerol, and 1 mM TCEP for 2 h to remove excess SAM, and then concentrated to 5 mg/mL with ultrafiltration. Then, the solution was flash-frozen in liquid nitrogen and stored at −20 °C.

### 3.4. Homogeneous Time-Resolved Fluorescent Energy Transfer (Htrf) Assay

MTase activity was determined with an EPIgeneous Methyltransferase Assay kit by assaying the conversion of SAM to SAH according to the manufacturer’s instructions. The enzymatic reaction was performed in white ProxiPlate-384 Plus microplates using a final reaction volume of 10 µL. The reaction buffer was composed of 40 mM Tris-HCl pH 8.3 (pH 8.0 for nsp16/nsp10) and 100 mM NaCl (or 10 mM KCl only for nsp16/nsp10), 1 mM DTT, 2 mM MgCl_2_, 0.01% Tween20. Then, 4 µL of purified recombinant protein nsp14 at 0.4 µM or nsp16/nsp10 at 3 µM final concentration were added in the assay wells, containing previously dispensed inhibitors. The reaction was started by preparing a mix containing 4 µM GpppG (Jena Bioscience, cat.nr. NU854) or ≈5 μM m7GpppA-RNA (for nsp16/nsp10) and SAM at 10 µM final concentrations in a volume of 4 µL, which was incubated at 37 °C for 20 min (2 h for nsp16/nsp10).

All compounds were dissolved in 100% DMSO (0.1% final DMSO concentration) and were tested at 100 µM concentration in duplicate. IC_50_ values were determined for compounds showing higher than 50% inhibitory effect at 100 µM concentration. For the detection of released SAH, 2 µL of EPIgeneous detection buffer one was added in order to stop the enzymatic reaction. After 10 min of incubation at room temperature, detection reagents were added: first, 4 µL of a 1/16 dilution of SAH-d2 conjugate and then 4 µL of Anti-SAH-Lumi4-Tb at a 1/100 dilution. HTRF signals were measured using a Hidex Sense (Finland) microplate reader using an excitation filter at 337 nm and fluorescence wavelength measurements at 620 and 665 nm, an integration delay of 150 ms, and an integration time of 400 ms. Results were analysed by calculating a two-wavelength signal ratio:

[intensity (665 nm)/intensity (620 nm)] × 104 (HTRF Ratio).(1)

The mean HTRF Ratio for each sample was calculated as:

Mean HTRF Ratio = Mean Sample HTRF Ratio − Blank HTRF Ratio,(2)

where ‘blank’ is the signal with the compound (or DMSO in control sample) and Anti-SAH-Lumi4-Tb. Percent inhibition was calculated using the following formula for each inhibitor dilution:

% Inhibition = 100 − (max signal comp − min signal comp) × 100/(max signal control − min signal control),(3)

where ‘max signal’ is the signal ratio without protein (negative control) and ‘min signal’ is the signal ratio in the sample. The IC_50_ value was calculated using the program Graph Pad Prism 8.0.

The selectivity of the compounds was tested using human Glycine N-Methyltransferase (GNMT from MyBioSource, cat. nr. MBS636160) using the same EPIgeneous Methyltransferase Assay kit. Reaction mixtures (volume 10 µL) contained 20 mM Tris-HCl (pH 8.6), 2 mM MgCl_2_, 0.5 µM GNMT, and compounds 1 nM to 10 µM. The reaction was started with a mix containing glycine and SAM at 5 mM and 10 µM final concentrations respectively and incubated at 25 °C for 30 min. Released SAH was detected, and the compound IC_50_ values were determined as described above.

### 3.5. SARS-CoV-2 Nsp16/Nsp10 Methyltransferase Substrate RNA Production

RNA oligonucleotide substrate for the nsp16/nsp10 assay was acquired through in vitro transcription with a Thermo Fisher TranscriptAid T7 High Yield Transcription Kit, using a DNA oligonucleotide Co25F 5′-TAC AAA GCT TCA GTA ATA CGA CTC ACT ATT ATA GAA CTT CGT CGA GTA CGC TCA A as a sense strand, and a DNA oligonucleotide Co25R 5′-TTG AGC GTA CTC GAC GAA GTT CTA TAA TAG TGA GTC GTA TTA CTG AAG CTT TGT A as a complimentary strand, to allow the transcription from the class II T7 promoter 5′- TAATACGACTCACTATTA 3′ and to obtain the RNA oligonucleotide m7G(5′)ppp(5′)Co25: m7G(5′)ppp(5′)AUAGAACUUCGUCGAGUACGCUCAA.

The in vitro transcription reaction was set up as follows:
DEPC-treated water6 µL5x TranscriptAid reaction buffer4 µLATP, Tris buffered, 30 mM1 µL (1.5 mM final concentration)CTP, Tris buffered, 100 mM1.5 µL (7.5 mM final concentration)GTP, Tris buffered, 100 mM1.5 µL (7.5 mM final concentration)UTP, Tris buffered, 100 mM1.5 µL (7.5 mM final concentration)Cap analog G(5′)ppp(5′)A, 100 mM1.2 µL (6 mM final concentration)Co25 template DNA, double stranded, preheated at 95 °C for 10 min, then slowly cooled down to 35 °C1 µg (0.5 µL)TranscriptAid enzyme mix2 µL

The reaction was incubated for 6 h at 37 °C, products were desalted via Thermo Scientific Zeba Spin Desalting Columns, 7K MWCO, 0.5 mL in DEPC-treated water, and then stored at −20 °C. A co-transcriptionally capped m7G(5′)ppp(5′)-Co25 oligonucleotide was obtained using 1.5 mM final concentration of ATP and 6.0 mM final concentration of G(5′)ppp(5′)A RNA Cap Structure Analog obtained from New England Bio Labs inc. Oligonucleotide was purified from template DNA by digestion with DNAse I for 15 min at 37 °C and subsequent desalting via Thermo Scientific Zeba Spin Desalting Columns, 7K MWCO, and 0.5 mL in DEPC-treated water. RNA oligonucleotide concentration was measured using a Nanodrop 2000 spectrophotometer, yielding concentrations of 2.5 ± 0.5 µg/µL and the oligonucleotides were analysed by 20% polyacrylamide denaturing (7 M urea) gel electrophoresis, where they appeared as a diffuse band around 8.3–8.6 kDa (Figure S1).

### 3.6. Cell Lines and Culture

A549 human non-small cell lung cancer cell line, HepG2 hepatocarcinoma cells, and BALB/c 3T3 clone A31 murine fibroblast cell line for the study were obtained from ATCC (American Type Culture Collection, Manassas, VA, USA). Cells were propagated in DMEM medium (Sigma, D6046, Irvin, UK) supplemented with 1% penicillin (100 U/mL)–streptomycin (100 μg/mL) and 10% foetal bovine serum (Sigma, F7524, St Louis, MO, USA) in case of A549 and HepG2 or 10% calf serum (Sigma, C8056, St Louis, MO, USA) in case of BALB/c 3T3. All cultivations were performed in a humidified 5% CO_2_ atmosphere at 37 °C.

### 3.7. Cytotoxicity Testing

The cytotoxicity of the compounds was tested in A549, HepG2, and BALB/c3T3 cell lines by neutral red (NR) uptake assay. A549 and BALB/c3T3 cells were seeded in 96-well plates at a density of 8 × 10^3^ cells per well, HepG2 at density 1.2 × 10^4^ cells per well. After 24 h incubation, test compounds in a concentration range of 0.8 to 200 μM were added. Cultivation in the presence of test compounds was done for 48 h. Afterwards, the plates were washed with phosphate-buffered saline (PBS) (Sigma, D8537, Irvin, UK), and 25 µg/mL NR solution (Sigma, N2889, Irvin, UK) was added. In case of BALB/c 3T3 cells, NR was diluted in 5% foetal calf serum containing media, while for A549 and HepG2 cells, it was diluted in 5% foetal bovine serum containing media. After 3 h incubation, the plate was washed with PBS, and the NR taken up by viable cells was extracted using desorbing fixative (50% ethanol/1% acetic acid/49% water). Absorbance at 540 nm was measured using a Tecan M200 Infinite Pro microplate reader. Cytotoxicity was expressed as a concentration-dependent reduction of the uptake of NR, compared to the untreated controls, and the IC_50_ value for each compound was calculated.

### 3.8. Cell Permeability Testing

#### 3.8.1. Compound Incubation in Cell Culture

To test cell permeability, each compound was added to A549 cell culture at a concentration of 20 μM and incubated for 24 h. Cells were seeded in 6-well plates at a density of 2 × 10^4^ and 4 × 10^4^ cells per well at concentrations 1 × 10^4^ and 2 × 10^4^ cells/mL media for the testing of each compound, each in three replicates. After the incubation, the cell cultivation media and cell lysates were collected. After the removal of media, cells were washed with ice-cold PBS (Sigma, D1408, Irvin, UK) and lysed for 30 min in 500 µL per well ice-cold RIPA buffer (Sigma, R0278, St Louis, MO, USA). Samples were stored at −80 °C until analysis.

#### 3.8.2. LC/MS/MS Analysis

The quantitative determination of tested compounds in cell lysates and culture media was performed on a Waters MICROMASS QUATTRO microTM tandem mass spectrometer combined with Acquity UPLC system. Acquity UPLC BEH C8 (2.1 × 50 mm × 1.7 μm) column was used at a flow rate of 0.25 mL/min. The column oven was set at 30 °C, and the sample injection volume was 5 μL. The mobile phase consisted of a combination of A (0.1% formic acid in water) and B (acetonitrile). The gradient elution program from 5% B to 98% B in 5 min was applied for analysis. Tandem mass spectrometer in positive electrospray mode was used for quantification. The TargetLynx software was applied to process LC/MS/MS data. Samples for LC/MS/MS analysis were prepared as follows: 200 μL of acetonitrile/methanol mixture (3:1 *v*/*v*) was add to 200 μL of each cell lysate and culture media sample. Then, the samples were centrifuged (10,000 rpm, 10 min), and the supernatants (300 μL) were removed from each sample and placed in vials for LC/MS/MS analysis. Calibration curves for all analytes were produced by analysis of standard solutions over a concentration range of 0.2 to 20 μM.

## 4. Conclusions

In conclusion, the adenosyl group containing analogues is a quick way to obtain potent SARS-CoV-2 MTase inhibitors; however, these compounds show poor selectivity and cell permeability. The identified drug-like nsp14 and nsp16 inhibitors also suffer from poor selectivity; however, the cell permeability of these compounds is slightly better. The compounds identified explore a wide range of chemotypes that interact with the adenine binding subsite and either the methionine or mRNA base binding sub-site of the MTases. Despite underwhelming selectivity, we believe that the identified inhibitors can serve as solid starting points for inhibitor development against the well-conserved nsp14 and nsp16 of SARS-CoVs.

## Figures and Tables

**Table 1:** Cytotoxicity and cell permeability of identified inhibitors and sinefungin.

Compound	Cytotoxicity (CC_50_), μM			Cell Permeability, %	
	3T3	HepG2	A549	2 × 10^4^ Cells/L	4 × 10^4^ Cells/L
HTVS drug-like hits					
ZINC38661771	115.6	93.84	96.96	<LOQ	<LOQ
ZINC23398144	>100	>100	>100	0.7	0.6
ZINC33037945	>100	>100	>100	17.9	30.8
SAM analogues					
ZINC4228245	>200	>200	>200	0.7	0.8
ZINC3861767	>200	>200	>200	2.2	0.5
ZINC473112262	>100	>100	>100	0.3	0.3
Sinefungin	99.21	>100	72.93	<LOD	<LOD

LOD—limit of detection; LOQ—limit of quantification (0.2 μM or 1%).

## Data Availability

The data presented in this study are available in article and Supplementary Material.

## Supplementary Materials

The following are available online at https://www.mdpi.com/article/10.3390/ph14121243/s1, Figure S1: Analysis of RNA transcripts by 20 % polyacrylamide denaturing gel electrophoresis, Table S1: The inhibition potency, ligand efficiency and target MTase of commercially obtained HTVS hits, Table S2: Enzymatic potency, ligand efficiency of commercially available ZINC23398144 hit analogues, Table S3: Enzymatic potency, ligand efficiency of commercially available ZINC38661771hit analogues, Table S4: Enzymatic potency and ligand efficiency of commercially available SAM analogues.
