# Supplementary material for: Discovery of SARS-CoV-2 Nsp14 and Nsp16 Methyltransferase Inhibitors by High-Throughput Virtual Screening
Source: Pharmaceuticals (Basel). 2021 Nov 30;14(12):1243. doi: 10.3390/ph14121243 (PMC8705538; doi:10.3390/ph14121243)
Supplement: Supplementary file 1 [file pharmaceuticals-14-01243-s001.zip › pharmaceuticals-1470720-supplementary.pdf]

# Discovery of SARS-CoV-2 nsp14 and nsp16 methyltransferase inhibitors by high-throughput virtual screening

Raitis Bobrovs,[†]* Iveta Kaņepe,[†] Nauris Narvaišs[†], Liene Patetko,[‡] Gints Kalniņš,[§] Mihails Šišovs,[§] Anna L. Bula,[†] Solveiga Grīnberga,[†] Mārtiņš Borodušķis,[‡] Anna Ramata-Stunda,[‡] Nils Rostoks,[‡] Aigars Jirgensons,[†] Kaspars Tārs,[§] Kristaps Jaudzems

[†]Latvian Institute of Organic Synthesis, Aizkraukles 21, Riga LV-1006, Latvia

[‡]University of Latvia, Jelgavas 1, Riga LV-1004, Latvia

[§]Latvian Biomedical Research and Study Centre, Ratsupites 1 k1, Riga LV-1067, Latvia

## Enzymatic assay data

Table S1. The inhibition potency, ligand efficiency and target MTase of commercially obtained HTVS hits

| No | Compound<br>(MolPort ID)<br>(ZINC ID) | Target MTase | Percent inhibition at 100 µM, %<br>($IC_{50}$, µM)<br>(Ligand Efficiency (LE), kcal/mol) | | |
|---|---|---|---|---|---|
| | | | nsp14 | nsp16 | Human |
| 1 | 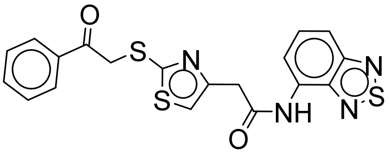<br>009-724-498<br>ZINC000038661771 | nsp14 | 58 ±7<br>$IC_{50}$=118 ±4<br>LE=0.19 | 69 ±8<br>$IC_{50}$=51 ±2<br>LE=0.21 | 63 ±5<br>$IC_{50}$=58 ±3<br>LE=0.21 |
| 2 | 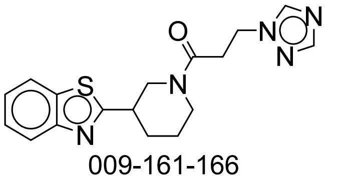<br>009-161-166<br>ZINC000023398144 | nsp14 | 57 ±8<br>$IC_{50}$=77 ±3<br>LE=0.23 | 39 ±7<br>$IC_{50}$=197 ±9<br>LE=0.21 | 21 ±2<br>$IC_{50}$=315 ±5<br>LE=0.20 |
| 3 | 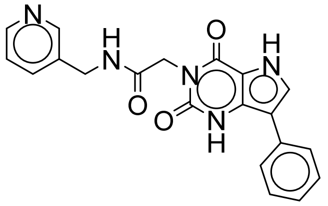<br>007-818-785<br>ZINC000033037945 | nsp14 | 56 ±5<br>$IC_{50}$=106 ±5<br>LE=0.19 | -8 ±6<br>inactive | 53 ±8<br>$IC_{50}$=125 ±6<br>LE=0.19 |

| | | | | | |
|---|---|---|---|---|---|
| 4 | 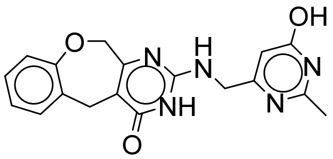<br>028-735-286*<br>ZINC000095519272 | nsp16 | 49 ±17<br>$IC_{50}$=~200<br>LE=0.19 | 57 ±13<br>$IC_{50}$=95 ±4<br>LE=0.21 | 70 ±3<br>$IC_{50}$=53 ±3<br>LE=0.22 |
| 5 | 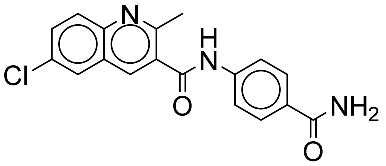<br>007-242-697<br>ZINC000019718669 | nsp14 | 49 ±16 | 66 ±9 | 52 ±4 |
| 6 | 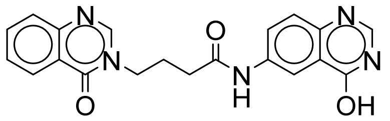<br>029-999-750<br>ZINC000096114839 | nsp14 | 28 ±7 | 17 ±5 | 83 ±6 |
| 7 | 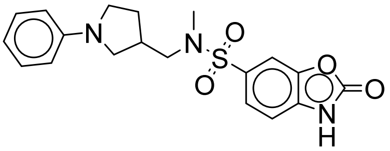<br>045-937-707<br>ZINC000952973746 | nsp14 | 27 ±9 | 38 ±6 | 41 ±1 |
| 8 | 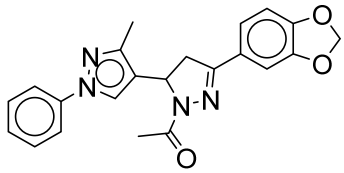<br>004-649-205<br>ZINC000012821397 | nsp14 | 27 ±5 | 15 ±5 | -5 ±2 |
| 9 | 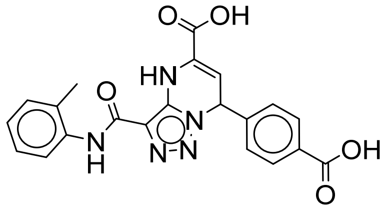<br>023-306-621<br>ZINC000072333096 | nsp14 | 25 ±10 | 33 ±12 | 17 ±4 |
| 10 | 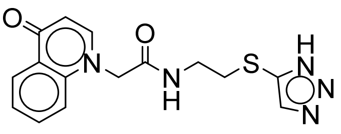<br>038-393-864<br>ZINC000218615582 | nsp16 | 21 ±3 | 51 ±4 | 55 ±2 |
| 11 | 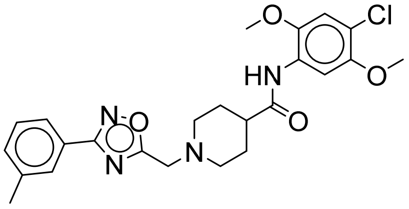<br>006-348-599<br>ZINC000030974583 | nsp16 | 19 ±1 | 45 ±11 | 48 ±8 |

| | | | | | |
|---|---|---|---|---|---|
| 12 | 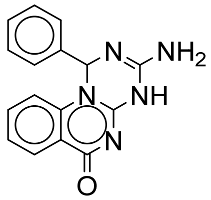 000-511-203 ZINC000008994271 | nsp16 | 19 ±10 | 39 ±3 | -3 ±6 |
| 13 | 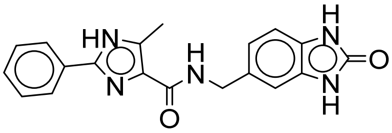 045-910-851 ZINC000680227150 | nsp14 | 19 ±7 | 23 ±5 | not tested |
| 14 | 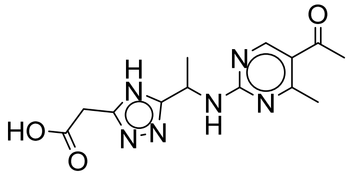 046-744-664 ZINC1772709012 | nsp16 | 16 ±13 | 32 ±8 | not tested |
| 15 | 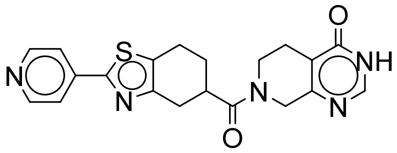 046-750-782 ZINC1772809556 | nsp14 | 15 ±9 | 5 ±8 | not tested |
| 16 | 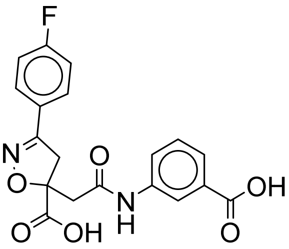 021-745-257 ZINC000072406141 | nsp14 | 14 ±7 | 31 ±8 | not tested |
| 17 | 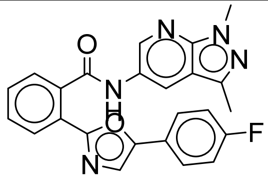 009-255-069 ZINC000032754717 | nsp14 | 14 ±6 | -24 ±9 | not tested |
| 18 | 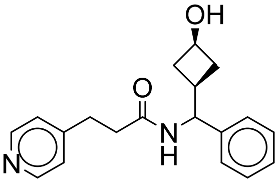 038-397-244 ZINC000252528037 | nsp14 | 13 ±2 | 36 ±3 | not tested |

| | | | | | |
|---|---|---|---|---|---|
| 19 | 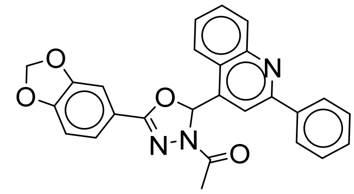 010-686-097 ZINC000021941344 | nsp14 | 12 ±4 | 36 ±10 | 45 ±7 |
| 20 | 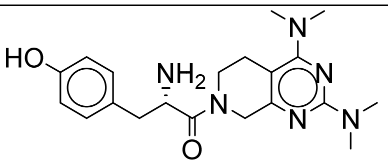 020-196-938 ZINC000071682020 | nsp14 | 12 ±5 | 9 ±8 | not tested |
| 21 | 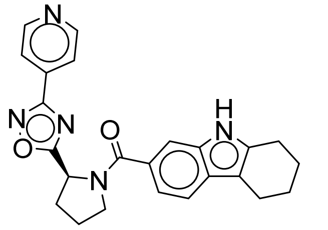 045-941-016 ZINC000952966781 | nsp14 | 11 ±10 | 24 ±5 | not tested |
| 22 | 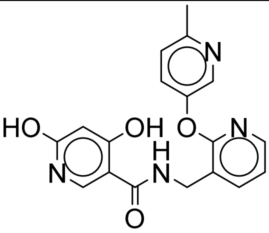 019-887-754 ZINC000067754517 | nsp16 | 11 ±3 | 39 ±8 | not tested |
| 23 | 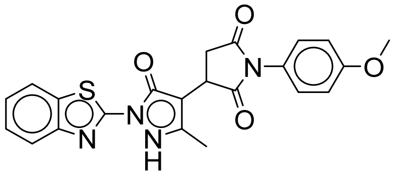 000-846-375 ZINC000018085229 | nsp14 | 11 ±6 | 17 ±6 | not tested |
| 24 | 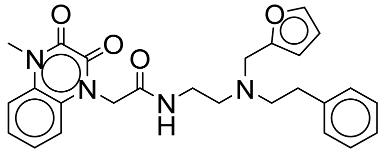 007-880-423 ZINC000021649685 | nsp16 | 10 ±9 | 7 ±4 | not tested |
| 25 | 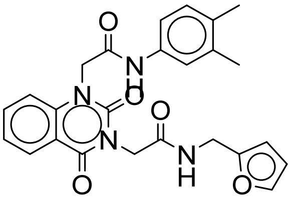 007-610-022 ZINC000009617688 | nsp14 | 10 ±6 | -8 ±4 | not tested |

| | | | | | |
|---|---|---|---|---|---|
| 26 | 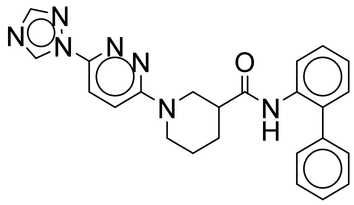 028-851-132 ZINC000096124096 | nsp14 | 9 ±7 | 10 ±1 | not tested |
| 27 | 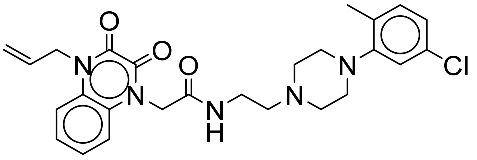 010-695-959 ZINC000033268761 | nsp16 | 9 ±3 | 32 ±7 | not tested |
| 28 | 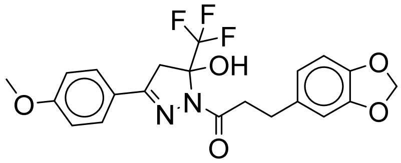 002-297-972 ZINC000016448507 | nsp14 | 9 ±4 | 11 ±5 | not tested |
| 29 | 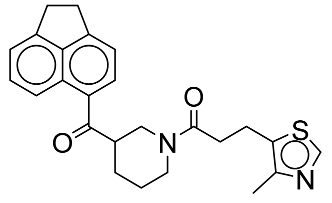 005-070-505 ZINC000012755219 | nsp14 | 9 ±5 | 40 ±8 | 61 ±4 |
| 30 | 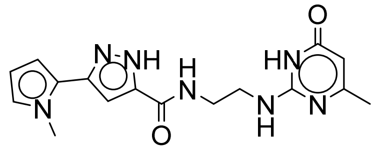 027-822-894 ZINC000091693861 | nsp16 | 9 ±6 | 24 ±4 | 21 ±6 |
| 31 | 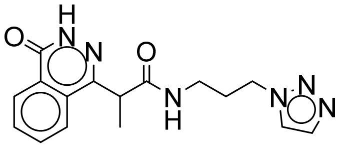 035-370-066 ZINC000215108606 | nsp16 | 9 ±3 | 40 ±6 | 22 ±6 |
| 32 | 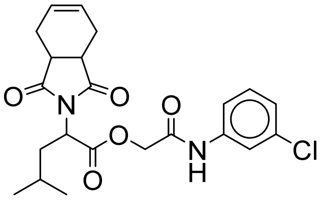 004-007-966 ZINC000019078629 | nsp14 | 8 ±3 | 37 ±9 | not tested |
| 33 | 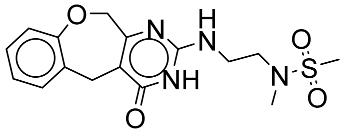 028-736-920 | nsp16 | 8 ±5 | -4 ±3 | 42 ±2 |

| | | | | | |
|---|---|---|---|---|---|
| | ZINC000095521577 | | | | |
| 34 | 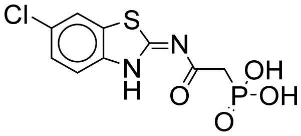 000-770-854 ZINC000020502712 | nsp16 | 7 ±6 | 17 ±7 | not tested |
| 35 | 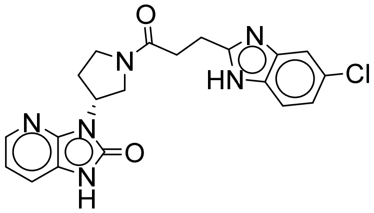 046-170-871 ZINC1548405210 | nsp14 | 6 ±2 | 15 ±2 | not tested |
| 36 | 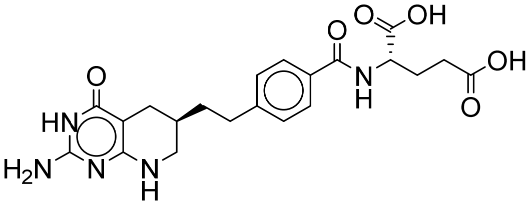 044-561-472 ZINC000077301924 | nsp14 | 5 ±6 | 15 ±10 | not tested |
| 37 | 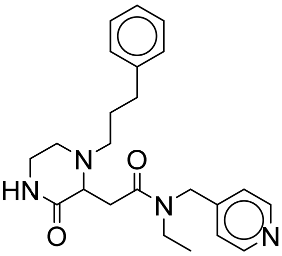 005-137-833 ZINC000011821342 | nsp14 | 5 ±5 | 17 ±11 | not tested |
| 38 | 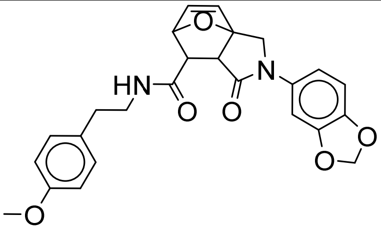 000-859-258 ZINC000012895535 | nsp14 | 5 ±2 | -15 ±6 | not tested |
| 39 | 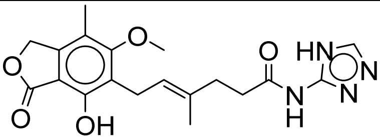 028-855-794 ZINC000096115284 | nsp16 | 5 ±4 | -68 ±6 | not tested |
| 40 | 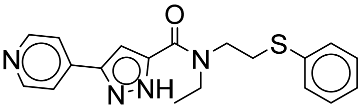 030-055-667 ZINC000097636636 | nsp14 | 5 ±2 | 19 ±7 | -46 ±2 |

| | | | | | |
|---|---|---|---|---|---|
| 41 | 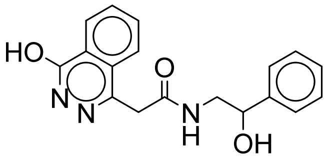 000-856-143 ZINC000009340377 | nsp16 | 5 ±4 | 2 ±5 | not tested |
| 42 | 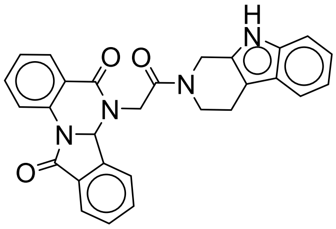 044-430-985 ZINC000604419204 | nsp14 | 5 ±11 | -3 ±5 | not tested |
| 43 | 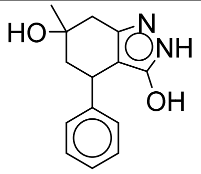 006-828-073 ZINC000035326963 | nsp16 | 4 ±8 | -2 ±6 | not tested |
| 44 | 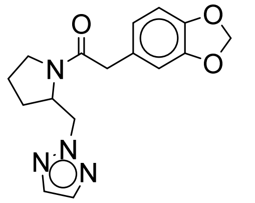 044-563-263 ZINC000019345667 | nsp14 | 4 ±1 | 22 ±6 | not tested |
| 45 | 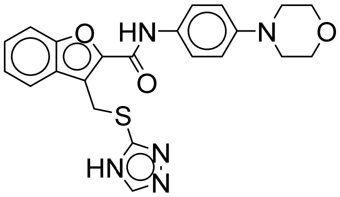 004-060-271 ZINC000003404544 | nsp14 | 3 ±2 | 6 ±8 | not tested |
| 46 | 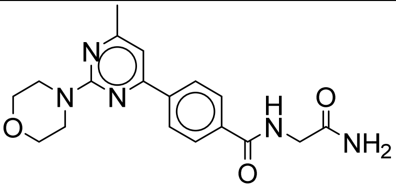 044-674-956 ZINC000534574506 | nsp16 | 3 ±3 | 17 ±8 | not tested |
| 47 | 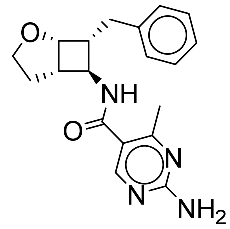 038-401-110 ZINC000219517301 | nsp14 | 3 ±4 | 28 ±8 | not tested |

| | | | | | |
|---|---|---|---|---|---|
| 48 | 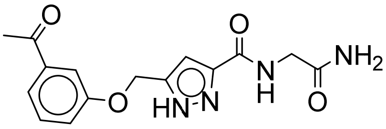 019-899-381 ZINC000067920483 | nsp16 | 2 ±2 | -1 ±3 | not tested |
| 49 | 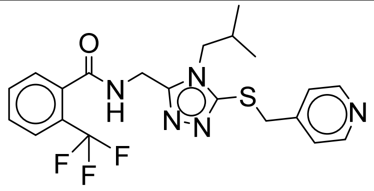 005-103-886 ZINC000012152850 | nsp16 | 2 ±8 | 19 ±6 | not tested |
| 50 | 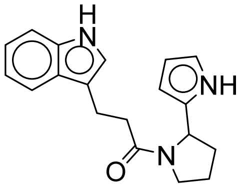 009-042-162 ZINC000019688789 | nsp14 | 2 ±0 | -10 ±1 | not tested |
| 51 | 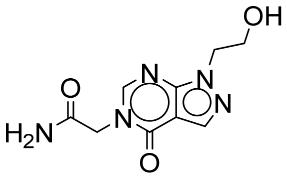 003-162-096 ZINC000004940485 | nsp16 | 2 ±0 | 6 ±6 | not tested |
| 52 | 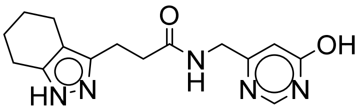 045-938-431 ZINC000952975967 | nsp16 | 2 ±7 | -24 ±4 | not tested |
| 53 | 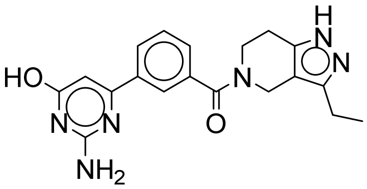 042-563-901 ZINC000426470786 | nsp14 | 1 ±6 | 8 ±5 | not tested |
| 54 | 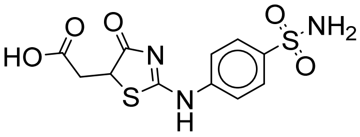 000-251-117 ZINC000001797008 | nsp16 | 0 ±2 | -35 ±10 | not tested |
| 55 | 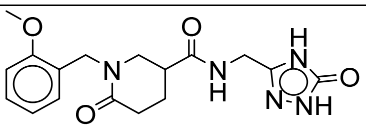 027-887-115 ZINC000092001674 | nsp16 | 0 ±7 | 22 ±12 | 44 ±8 |

| | | | | | |
|---|---|---|---|---|---|
| 56 | 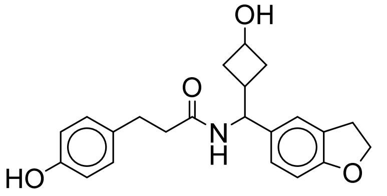 038-410-201 ZINC000220681335 | nsp14 | 0 ±2 | 10 ±5 | not tested |
| 57 | 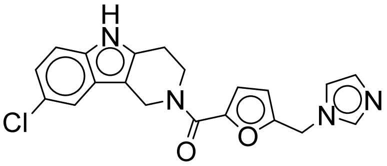 021-759-980 ZINC000072429175 | nsp14 | 0 ±4 | 6 ±5 | not tested |
| 58 | 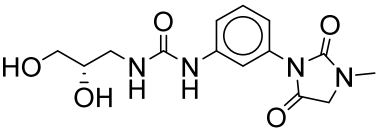 042-578-273 ZINC000426716892 | nsp16 | -1 ±2 | 30 ±2 | not tested |
| 59 | 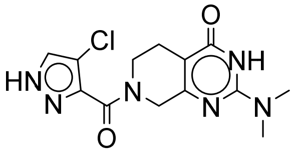 020-232-114 ZINC000072172639 | nsp16 | -1 ±2 | 29 ±5 | not tested |
| 60 | 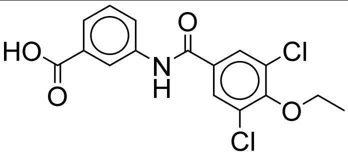 002-245-502 ZINC000004649202 | nsp16 | -1 ±3 | 14 ±5 | not tested |
| 61 | 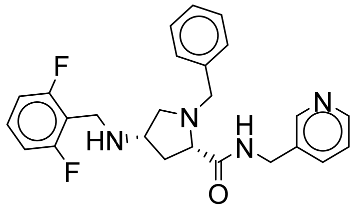 005-074-359 ZINC000020999776 | nsp14 | -2 ±4 | 8 ±5 | not tested |
| 62 | 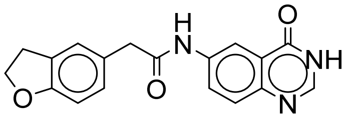 035-814-299 ZINC000170622294 | nsp16 | -2 ±2 | -6 ±3 | not tested |
| 63 | 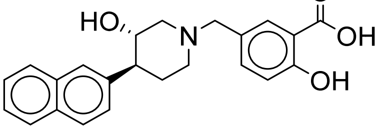 016-617-317 ZINC000065452571 | nsp14 | -3 ±8 | -2 ±5 | not tested |

| | | | | | |
|---|---|---|---|---|---|
| 64 | 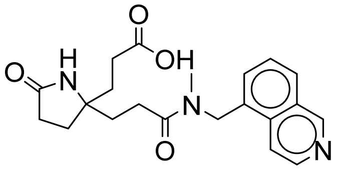 038-408-691 ZINC000220483024 | nsp16 | -5 ±3 | 31 ±6 | 16 ±4 |
| 65 | 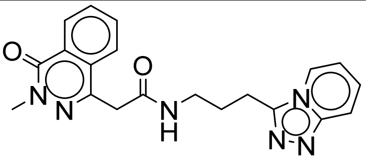 028-605-864 ZINC000095478169 | nsp16 | -6 ±2 | -53 ±8 | not tested |
| 66 | 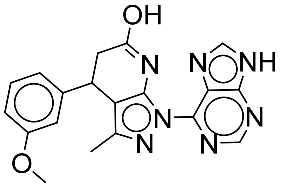 006-807-837 ZINC000020745418 | nsp14 | -8 ±5 | -7 ±2 | not tested |
| 67 | 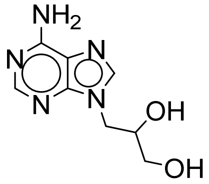 001-002-015 ZINC000000370772 | nsp16 | -9 ±12 | -34 ±9 | not tested |
| 68 | 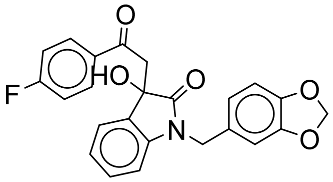 001-824-709 ZINC000000783765 | nsp16 | -10 ±3 | -3 ±4 | not tested |
| 69 | 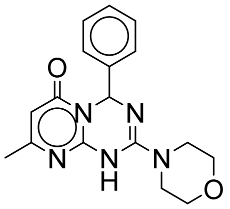 002-997-525 ZINC000104279901 | nsp16 | -14 ±2 | -33 ±4 | not tested |
| 70 | 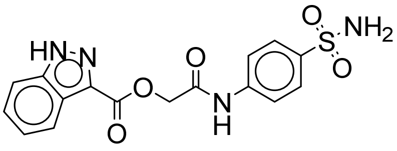 005-328-022 ZINC000005360401 | nsp16 | -15 ±7 | 3 ±1 | not tested |

| | | | | | |
|---|---|---|---|---|---|
| 71 | 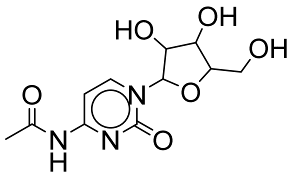 000-395-882 ZINC000001078602 | nsp16 | -15 ±5 | -9 ±1 | not tested |
| 72 | 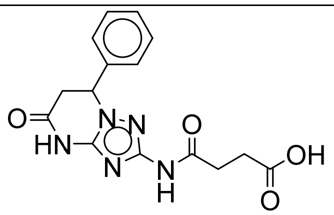 002-580-688 ZINC000000095711 | nsp16 | -16 ±0 | -77 ±13 | not tested |
| 73 | 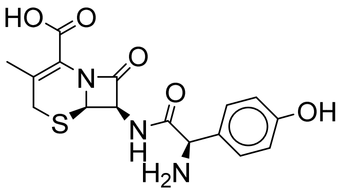 002-507-346 ZINC000003830391 | nsp16 | -18 ±6 | -8 ±1 | not tested |
| 74 | 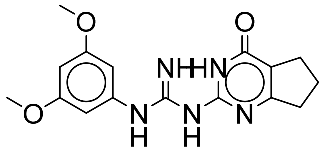 002-997-627 ZINC000009935252 | nsp16 | not tested** | not tested** | not tested** |
| 75 | 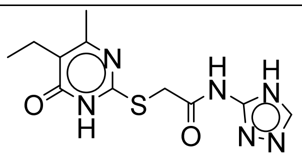 007-980-293 ZINC000033175254 | nsp16 | not tested** | not tested** | not tested** |
| 76 | 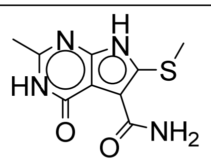 035-884-006 ZINC000006694156 | nsp16 | not tested** | not tested** | not tested** |
| 77 | 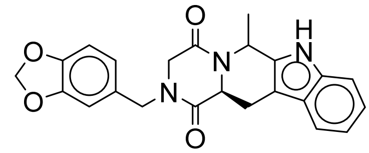 001-737-468 ZINC000002102009 | nsp16 | not tested** | not tested** | not tested** |
| 78 | 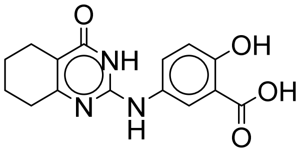 021-780-054 ZINC000073647922 | nsp16 | not tested** | not tested** | not tested** |

| | | | | | |
|---|---|---|---|---|---|
| 79 | 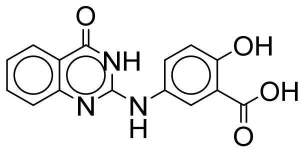<br>021-780-059<br>ZINC000073647929 | nsp16 | not tested** | not tested** | not tested** |
| 80 | 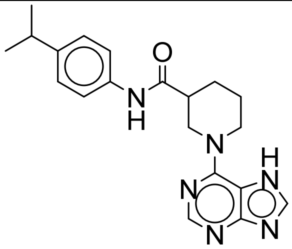<br>000-842-477<br>ZINC000009059943 | nsp14 | not tested** | not tested** | not tested** |
| 81 | 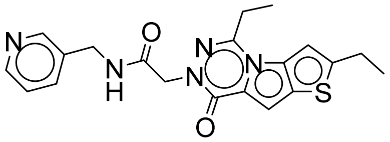<br>007-669-585<br>ZINC000012088756 | nsp14 | not tested** | not tested** | not tested** |

* NMR and MS analysis indicated that sample contains mixture of compounds

** compound not soluble in DMSO

Table S2. Enzymatic potency, ligand efficiency of commercially available ZINC23398144 hit analogues

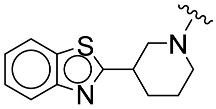

| | | Percent inhibition at 100 µM, % ($IC_{50}$, µM) (Ligand Efficiency (LE), kcal/mol) | | |
|---|---|---|---|---|
| No | Compound (MolPort ID) (ZINC ID) | nsp14 | nsp16 | Human |
| 1 | 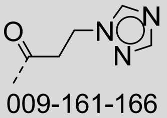 009-161-166 ZINC000023398144 | 57 ±8 $IC_{50}$=77 ±3 LE=0.23 | 39 ±7 $IC_{50}$=197 ±9 LE=0.21 | 21 ±2 $IC_{50}$=315 ±5 LE=0.20 |
| 2 | 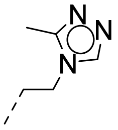 046-188-339 | 31 ±3 | 66 ±12 | not tested |
| 3 | 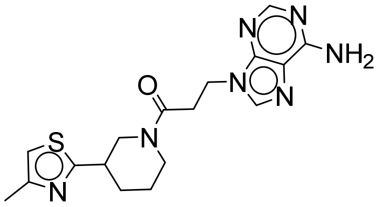 046-114-939 | 15 ±4 | 37 ±13 | not tested |
| 4 | 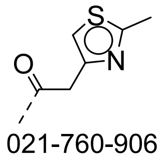 021-760-906 | 10 ±2 | 28 ±6 | not tested |
| 5 | 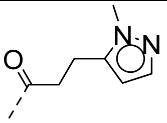 042-575-680 | 8 ±1 | 33 ±9 | not tested |
| 6 | 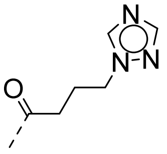 045-938-897 | 7 ±2 | -29 ±5 | not tested |
| 7 | 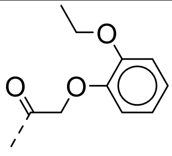 005-679-672 | 6 ±0 | -59 ±2 | not tested |
| 8 | 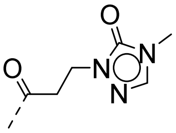 | 5 ±3 | 15 ±8 | not tested |

| | | | | |
|---|---|---|---|---|
| | 046-705-372 | | | |
| 9 | 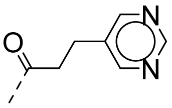 046-723-388 | 5 ±3 | -45 ±13 | not tested |
| 10 | 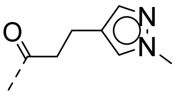 046-706-934 | 5 ±3 | -113 ±16 | not tested |
| 11 | 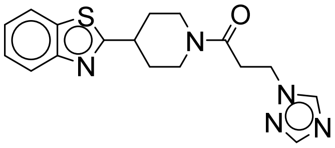 009-436-708 | 2 ±1 | -114 ±6 | not tested |
| 12 | 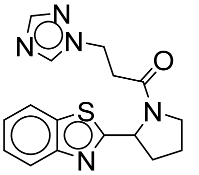 009-447-345 | 2 ±0 | 1 ±1 | not tested |

Table S3. Enzymatic potency, ligand efficiency of commercially available ZINC38661771hit analogues

| No | Compound (MolPort ID) (ZINC ID) | Percent inhibition at 100 µM, % ($IC_{50}$, µM) (Ligand Efficiency (LE), kcal/mol) nsp14 | nsp16 | Human |
|---|---|---|---|---|
| 1 | 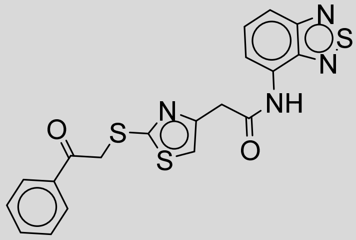 009-724-498 ZINC000038661771 | 58 ±7 $IC_{50}$=118 ±4 LE=0.19 | 69 ±8 $IC_{50}$=51 ±2 LE=0.21 | 63 ±5 $IC_{50}$=58 ±3 LE=0.21 |
| 2 | 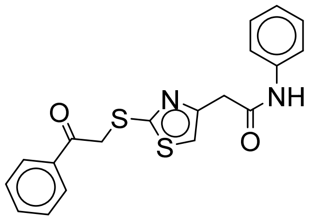 005-296-365 | 27 ±2 | -17 ±4 | not tested |
| 3 | 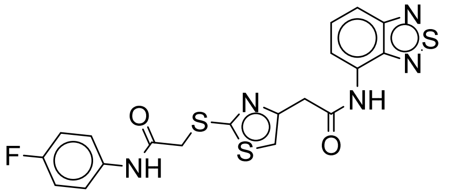 009-741-602 | 25 ±2 | 45 ±5 | 24 ±7 |
| 4 | 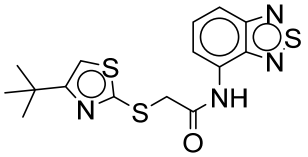 009-639-090 | 16 ±1 | 43 ±7 | 61 ±9 |

* Only 3 ZINC38661771 analogues were purchased due to limited chemical space covered by commercially available compounds – majority of analogues varied with the placement of minor functional groups, mostly halogen atoms, at the terminal benzene ring.

Table S4. Enzymatic potency and ligand efficiency of commercially available SAM analogues

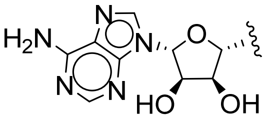


| No | Compound (MolPort ID) (ZINC ID) | Percent inhibition at 100 µM, % ($IC_{50}$, µM) (Ligand Efficiency (LE), kcal/mol) | | |
|---|---|---|---|---|
| | | nsp14 | nsp16 | Human |
| 1 | 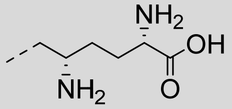 Sinefungin 044-183-244 ZINC000004217451 | $IC_{50}$=0.45 ±0.02 LE=0.32 | $IC_{50}$=0.86 ±0.04 LE=0.30 | $IC_{50}$=316 ±15 LE=0.18 |
| 2 | 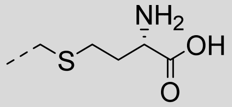 S-(5′-Adenosyl)-L-homocysteine (SAH) 003-925-198 ZINC000004228232 | $IC_{50}$=0.14 ±0.01 LE=0.33 | $IC_{50}$=0.28 ±0.03 LE=0.32 | $IC_{50}$=1.7 ±0.3 LE=0.28 |
| 3 | 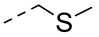 001-834-574 ZINC000004228245 | $IC_{50}$=1.4 ±0.1 LE=0.40 | $IC_{50}$=1.02 ±0.05 LE=0.41 | $IC_{50}$=1.8 ±0.2 LE=0.39 |
| 4 | 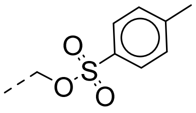 004-959-541 ZINC000003861767 | $IC_{50}$=1.5 ±0.2 LE=0.27 | $IC_{50}$=1.14 ±0.06 LE=0.28 | $IC_{50}$=2.80 ±0.14 LE=0.26 |
| 5 | 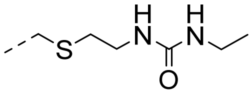 046-857-324 ZINC000473112262 | $IC_{50}$=3.1 ±0.3 LE=0.28 | $IC_{50}$=2.46 ±0.12 LE=0.28 | IC50=5.8 ±0.3 LE=0.26 |
| 6 | 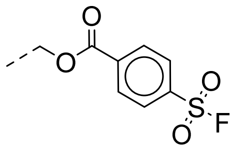 003-934-134 ZINC000048222133 | 58 ±2 $IC_{50}$=53 ±3 LE=0.19 | 42 ±6 $IC_{50}$=105 ±6 LE=0.17 | 87 ±1 $IC_{50}$=25.3 ±1.3 LE=0.20 |
| 7 | 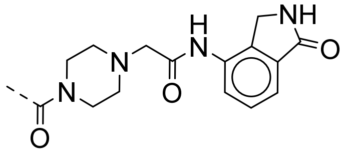 047-543-936 ZINC000017654909 | 62 ±2 $IC_{50}$=75 ±4 LE=0.14 | 65 ±7 $IC_{50}$=75 ±4 LE=0.14 | 98 ±2 $IC_{50}$=22.8 ±1.2 LE=0.16 |
| 8 | 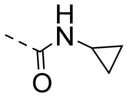 | 32 ±6 | 54 ±10 | 56 ±1 |

| | | | | |
|---|---|---|---|---|
| | 039-336-807<br>ZINC000004475116 | | | |
| 9 | 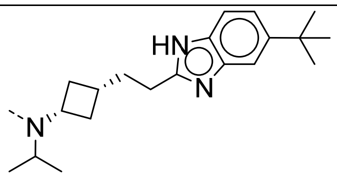<br>035-706-611<br>ZINC000100074350 | 30 ±4 | 68 ±7 | not tested |
| 10 | 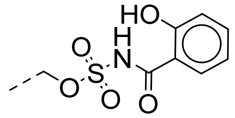<br>046-417-911<br>ZINC000028567519 | 28 ±2 | 14 ±3 | not tested |
| 11 | 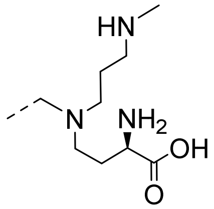<br>046-594-122<br>ZINC000584905214 | 22 ±5 | 1 ±5 | not tested |
| 12 | 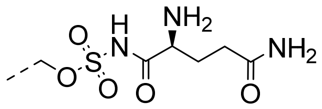<br>046-683-328<br>ZINC000036477981 | 20 ±3 | 50 ±11 | 89 ±8 |
| 13 | 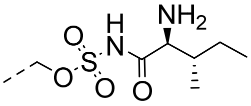<br>046-416-871<br>ZINC000013488353 | 8 ±5 | 34 ±6 | not tested |

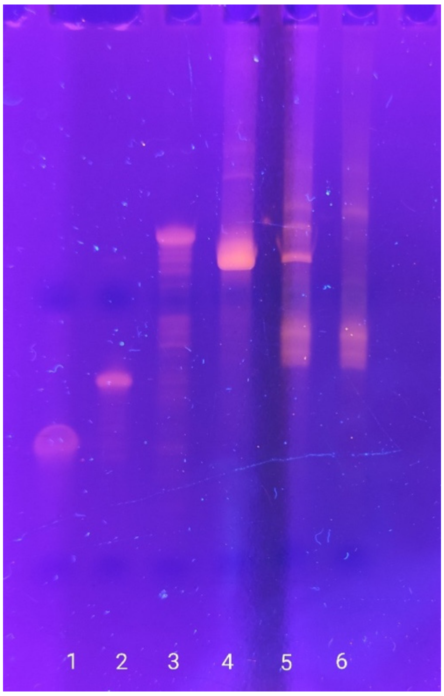


Figure S1. Analysis of RNA transcripts by 20 % polyacrylamide denaturing gel electrophoresis.

1. – 5.8 kDa standard

2. – 8.3 kDa

3. – 19.3 kDa

4. – DNA template

5. - m7G(5')ppp(5')Co25 co-transcriptionally capped RNA, before DNAse digestion

6. - m7G(5')ppp(5')Co25 co-transcriptionally capped RNA, after DNAse digestion, desalted
